# The virion-associated incoming HIV-1 RNA genome is not targeted by RNA interference

**DOI:** 10.1186/1742-4690-3-57

**Published:** 2006-09-04

**Authors:** Ellen M Westerhout, Olivier ter Brake, Ben Berkhout

**Affiliations:** 1 Laboratory of Experimental Virology, Department of Medical Microbiology, Center for Infection and Immunity Amsterdam (CINIMA), Academic Medical Center, University of Amsterdam, The Netherlands

## Abstract

**Background:**

RNA interference (RNAi) has proven to be a powerful tool to suppress gene expression and can be used as a therapeutic strategy against human pathogenic viruses such as human immunodeficiency virus type 1 (HIV-1). Theoretically, RNAi-mediated inhibition can occur at two points in the replication cycle, upon viral entry before reverse transcription of the RNA genome, and on the newly transcribed viral RNA transcripts. There have been conflicting results on whether RNAi can target the RNA genome of infecting HIV-1 particles. We have addressed this issue with HIV-1-based lentiviral vectors.

**Results:**

We determined the transduction efficiency of a lentiviral vector, as measured by GFP expressing cells, which reflects the number of successful integration events in a cell line stably expressing shNef. We did not observe a difference in the transduction efficiency comparing lentiviral vectors with or without the Nef target sequence in their genome. The results were similar with particles pseudotyped with either the VSV-G or HIV-1 envelope. Additionally, no reduced transduction efficiencies were observed with multiple other shRNAs targeting the vector genome or with synthetic siNef when transiently transfected prior to transduction.

**Conclusion:**

Our findings indicate that the incoming HIV-1 RNA genome is not targeted by RNAi, probably due to inaccessibility to the RNAi machinery. Thus, therapeutic RNAi strategies aimed at preventing proviral integration should be targeting cellular receptors or co-factors involved in pre-integration events.

## Background

Double stranded RNA (dsRNA) can induce RNA interference (RNAi) in cells, resulting in sequence-specific degradation of the targeted mRNA [1,2]. Short interfering RNAs (siRNAs) of ~22 nt are the effector molecules of this evolutionarily conserved mechanism and are produced by a ribonuclease named Dicer [3,4]. One strand of the siRNA duplex is incorporated into the RNA-induced silencing complex (RISC), which binds to and cleaves complementary RNA sequences [5,6]. RNAi has proven to be a powerful tool to suppress gene expression. Transfection of synthetic siRNA into cells results in transient inhibition of the targeted gene [7]. Stable gene suppression can be achieved by the introduction of vectors that express siRNAs or short hairpin RNAs (shRNAs) that are processed into siRNAs by Dicer [8,9].

RNAi can be used as a therapeutic strategy against human pathogenic viruses such as HIV-1 [10]. Several studies have demonstrated that HIV-1 replication can be inhibited transiently by transfection of synthetic siRNAs targeting either viral RNA sequences or cellular mRNAs encoding protein co-factors that support HIV-1 replication [11-20]. Furthermore, several groups have demonstrated long-term inhibition of HIV-1 replication in transduced cell lines that stably express an antiviral siRNA or shRNA [21-28]. However, HIV-1 escape variants with nucleotide substitutions or deletions in the siRNA target sequence emerge after prolonged culturing [22,24]. We have also demonstrated that HIV-1 can gain resistance against RNAi through mutations that mask the target in a stable RNA secondary structure [29]. The use of combination-shRNA therapy, in which multiple conserved viral RNA sequences are targeted by multiple shRNAs at the same time, may block the emergence of RNAi resistant variants [30].

During the HIV-1 life cycle, there are two phases that could potentially be targeted by RNAi [31,32]. Newly made viral transcripts, synthesized from the integrated proviral DNA, are the obvious targets. In addition, RNAi may target the virion-associated or "incoming" viral RNA genome during the initial phase of infection prior to completion of reverse transcription that converts the RNA genome into DNA. During the infection, the HIV-1 core particle traverses through the cytoplasm, where the RNAi machinery resides. If the RNA genome within the virion core is accessible to the RISC complex, reverse transcription and subsequent proviral integration would be blocked, which is highly desirable in a therapeutic setting. There have been conflicting results on whether RNAi can target the RNA genome of infecting HIV-1 particles. Several groups have reported degradation of the incoming RNA genome in cells transfected with siRNAs [11,12,16]. Recently, a study showed inhibition of HIV-1 provirus integration in cells stably expressing shRNAs at a low virus input [33]. Other publications report no RNAi-mediated degradation of the RNA genome in siRNA-transfected or shRNA-producing cells [17,18,34]. In the present study, we have readdressed the issue of incoming HIV-1 genome targeting using HIV-1-based lentiviral vectors in which we used transduction as a model for proviral integration. Targeting of the incoming genome did not reduce the transduction efficiency, indicating that the HIV-1 RNA genome is not a target for RNAi during the initial phase of infection.

## Results

**Figure 1:**
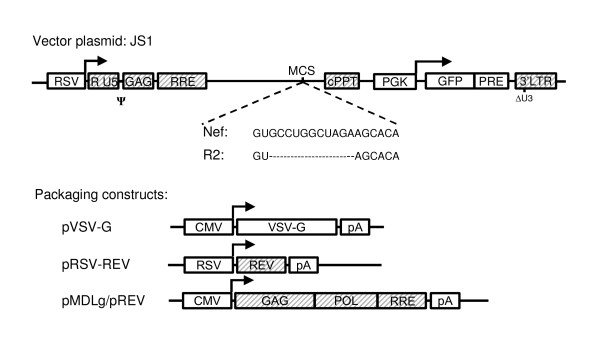
**The lentiviral vector and packaging constructs**. The lentiviral vector JS1 is a third generation self-inactivating vector [39], which contains a GFP reporter gene expressed from the phosphoglycate kinase promoter (PGK) with the posttranscriptional regulatory element (pre) from hepatitis B virus. The vector genome is expressed from the Rous sarcoma promoter (RSV) and transcription starts with the R and U5 regions of the HIV-1 long terminal repeat (LTR), the packaging signal (ψ) and part of the gag open reading frame (gag). It contains the rev responsive element (RRE), central polypurine tract (cPPT) and the 3' LTR, which has a deletion in the U3 region (ΔU3). The HIV-1 sequences are tinted gray. Transcription of the vector genome and GFP reporter terminates at the HIV-1 polyA within the 3'LTR. The Nef target sequence (wild type or mutant) was cloned into the multiple cloning site (MCS). The three packaging constructs encode the trans-acting proteins required for the production of infectious virus (HIV-1 sequences in gray).

To determine the amount of incoming HIV-1 RNA in cells expressing antiviral siRNAs, the integrated HIV-1 DNA product or pre-integration DNA intermediates have been quantified [12,16-18,33,34]. Instead, we use an HIV-1 based lentiviral vector system to study proviral integration in cells expressing shRNAs against the HIV-1 lentiviral vector genome. We chose the lentiviral vector system because it is ideally suited to study proviral integration since viral infection is limited to a single cycle and is easily scored with FACS analysis detecting reporter gene expression in transduced cells. JS1 is a third generation self-inactivating lentiviral vector containing a GFP reporter gene (Fig. 1). Lentiviral vector particles are produced in 293T cells by co-transfection of the vector plasmid with the packaging constructs encoding Gag-Pol, Rev, and the VSV-G envelope protein (Fig. 1). Transduction titers of the produced lentiviral vectors were determined. All infection experiments were subsequently carried out at relatively low multiplicity of infection (m.o.i) such that transduced cells were preferably infected by a single vector. Thus, a transduced cell represents a single successful reverse transcription and proviral integration event.

**Figure 2:**
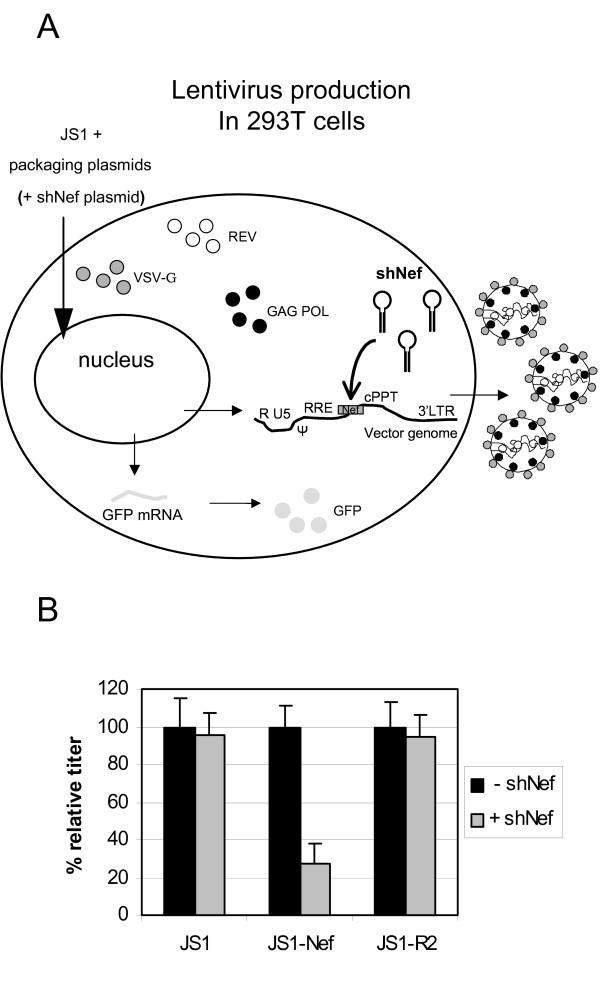
**Sequence-specific inhibition of lentiviral production by RNAi. a) **Schematic of lentiviral production. When an shNef-expression plasmid is co-transfected during lentiviral vector production, the lentiviral vector RNA genome containing the Nef target (gray box) can be targeted by RNAi (dark arrow). **b) **Lentiviral vector stocks (JS1, JS1-Nef and JS1-R2) were produced in 293T cells in the absence (-shNef) or presence (+shNef) of an shNef-expression plasmid and were titrated on SupT1 cells. Transduced cells were analyzed by GFP-FACS. The mean values of three independent experiments are shown. The control values (-shNef) were set at 100% for each lentiviral vector.

We cloned an approximately 200 bp Nef fragment into the multiple cloning site (MCS) of the lentiviral vector genome (JS1-Nef). This sequence contains the target sequence for the potent shNef inhibitor that we described in earlier studies [24,29]. As a control, we constructed a vector with a mutant Nef sequence (JS1-R2), lacking 11 nucleotides of the shNef target sequence, which was shown to be completely resistant to shNef attack [24,29]. During lentiviral vector production, the vector genome is transcribed and transported to the cytoplasm where it becomes packaged in the vector particle (Fig. 2a). When the JS1-Nef lentiviral particles were produced in the presence of the shNef expression plasmid in the transfection mix, we observed a significant reduction in titer (Fig. 2b). In contrast, the titer of JS1 and JS-R2 vectors was similar to their titer produced in the absence of shNef. This result shows that the vector genome is in principle an effective target for RNA interference.

**Figure 3:**
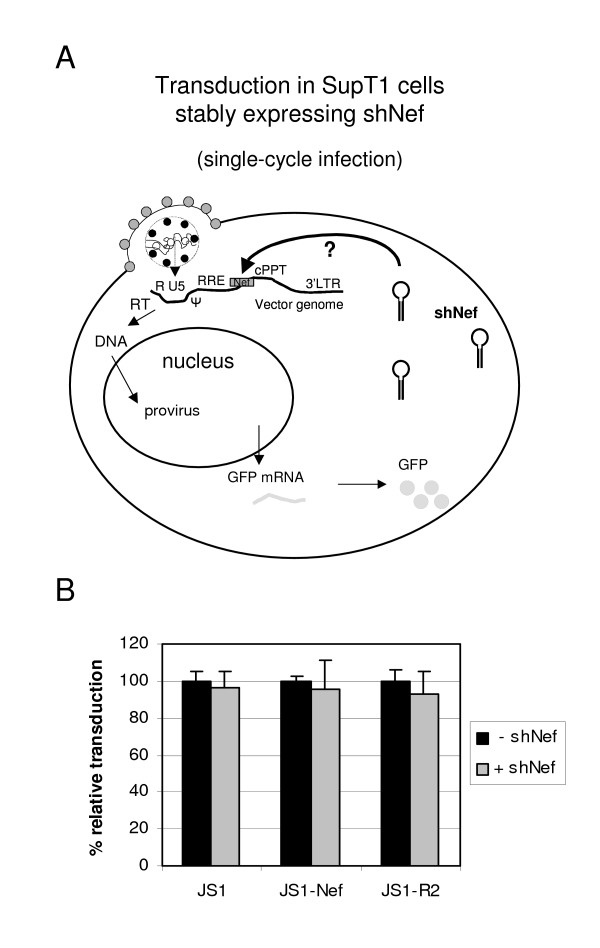
**No sequence-specific inhibition of lentiviral transduction by RNAi. a) **Schematic of lentiviral transduction. When shNef is stably produced in the target cells, the question is whether the incoming vector genome with the shNef target sequence is targeted by RNAi (dark arrow with question mark). **b) **SupT1 cells stably expressing shNef (+ shNef) or control SupT1 cells (- shNef) were transduced at an m.o.i. of 0.03, 0.3 or 1.0 with the control vector (JS1) or vectors containing a complete (JS1-Nef) or mutated (JS1-R2) shNef target sequence. Infected cells were analyzed by GFP-FACS. The control values (- shNef) were set at 100% for each lentiviral vector. The mean values of three experiments are shown.

The lentiviral vectors JS1, JS1-Nef and JS1-R2 were produced and subsequently used to infect the SupT1 T cell line that stably expresses shNef [24] and control SupT1 cells. When the incoming RNA genome is targeted by shRNA induced RNAi, the number of cells that obtain an integrated proviral DNA copy should be reduced. This will be reflected in a reduced transduction efficiency of shNef cells compared to the control SupT1 cells (Fig. 3a). Two days after infection, the cells were analyzed by FACS analysis. We did not observe a significant difference in the transduction efficiency of JS1-Nef in the control cells versus shNef-expressing cells, indicating that the incoming vector genome was not targeted by RNAi (Fig. 3b). Results were similar for the empty vector JS1 and control vector JS1-R2 with a deletion in the shNef target sequence. The results were independent of the m.o.i., which ranged from 0.03 to 1. These combined results clearly indicate that the incoming lentiviral RNA genome is not a target for RNAi.

**Figure 4:**
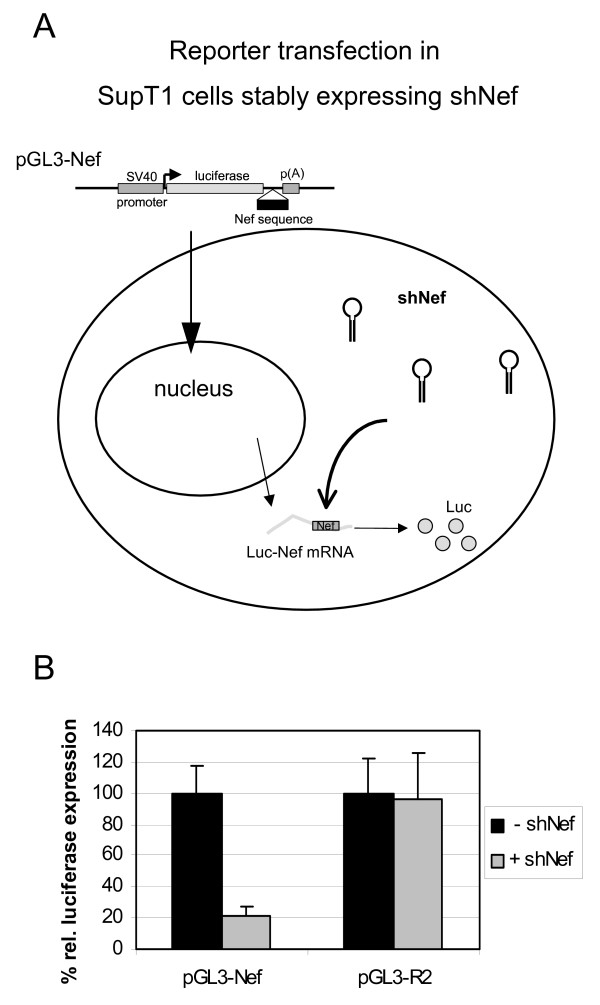
**Sequence-specific inhibition in shNef-expressing cells. a) **Schematic of RNAi-mediated targeting of mRNA with the shNef target sequence (gray box) in shNef-expressing SupT1 cells. **b) **SupT1 cells stably expressing shNef (+ shNef) or control SupT1 cells (- shNef) were transfected with luciferase reporter constructs that contain the complete shNef target sequence (pGL3-Nef) or not (pGL3-R2). The mean values obtained in two independent experiments are shown. Values measured in the control transfection (- shNef) were set at 100% for each reporter construct.

As an additional control for the presence of a functional shNef in the shNef-expressing SupT1 cells, we transfected the luciferase reporter constructs [29] containing the complete (pGL3-Nef) or mutant (pGL3-R2) target sequence (Fig. 4a). Luciferase expression of pGL3-Nef was reduced to 20% in the shNef-expressing cells compared to the control cells (Fig. 4b). In contrast, luciferase expression of pGL3-R2 is similar in both cells. This confirms that SupT1 cells expressing shNef induce sequence-specific inhibition of RNAs containing the Nef target sequence.

**Figure 5:**
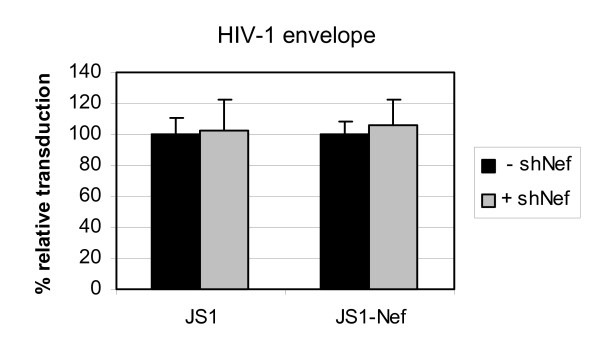
**No inhibition of lentiviral transduction with virions containing the HIV-1 Envelope**. SupT1 cells stably expressing shNef (+ shNef) or control SupT1 cells (- shNef) were transduced at an m.o.i. of 0.03, 0.2 or 0.5 with either the control (JS1) or the shNef target sequence containing (wt-Nef) lentiviral vector with an HIV-1 envelope protein. Infected cells were analyzed by GFP-FACS. The control values (- shNef) were set at 100% for each infection. The mean values of two independent experiments are shown.

The lentiviral particles used in the experiments described above are pseudotyped with the VSV-G envelope. One could argue that VSV-G mediated entry and subsequent intracellular processes are different from wildtype HIV-1 virions that contain the HIV-1 Envelope protein. The use of VSV-G would thus explain why we do not observe targeting of the incoming genome. To exclude this possibility, we produced lentiviral vectors with an HIV-1 Envelope and repeated the experiment. Infection of SupT1 cells expressing shNef with JS1-Nef lentivirus containing HIV-1 envelope was similar to that of control SupT1 cells, which demonstrates that the mode of entry does not contribute to the absence of incoming genome targeting (Fig. 5).

**Figure 6:**
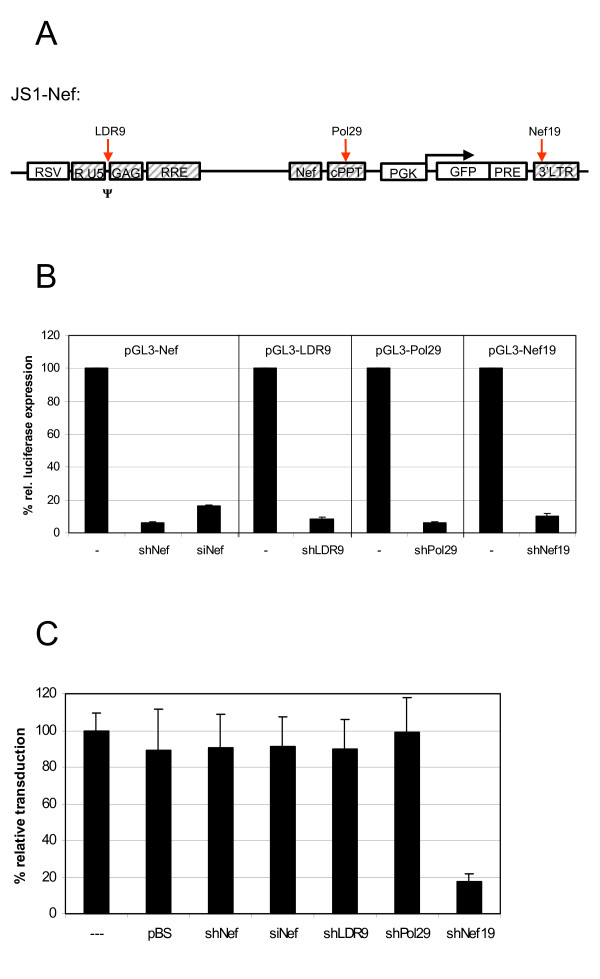
**No inhibition of lentiviral transduction in cells transfected with different shRNA plasmids or siRNA. a) **Map of the JS1-Nef genome with the positions targeted by the shRNA inhibitors. **b) **293T cells were mock transfected (-) or transfected with siNef or plasmids expressing the indicated shRNAs. The cells were subsequently transfected with luciferase reporter constructs containing the target sequences and relative luciferase expression was measured. The mean values obtained in two independent experiments are shown. The control value (-) was set at 100% for each luciferase reporter. **c) **293T cells were mock transfected (-) or transfected with the control pBS, siNef or plasmids expressing the indicated shRNA. The cells were subsequently transduced with the JS1-Nef vector. Transduction efficiency was determined by GFP-FACS. The mean values obtained in two independent experiments are shown. The transduction efficiency for the control experiment (-) was set at 100%.

The contradicting results in literature on inhibition of the incoming HIV-1 RNA genome by RNAi may be due to differences in experimental conditions. In fact, most studies used chemically synthesized siRNAs that were transfected into various cell types prior to challenge with HIV-1. We therefore tested a synthetic siRNA directed against the same shNef target. This siNef is the same as the one shown by Jacque et al. to affect the level of integrated provirus [12]. Cells transfected with siNef or a shNef expression plasmid reduced pGL3 Luciferase Nef reporter expression, when the reporter was transfected 24 hours post si or shRNA transfection (Fig. 6b). In contrast, when these siRNA or shRNA-expressing cells were infected with JS1-Nef lentiviral particles, no drop in transduction efficiency was observed compared to mock (-) or pBS-transfected cells (Fig. 6c). Similar results were obtained with a range of m.o.i. (results not shown). Thus, an active siRNA is also unable to inhibit the incoming RNA genome.

In literature, a variety of different targets have been used and variation in target accessibility in the context of the packaged RNA genome may explain the contradicting results. Our lab has constructed multiple potent shRNAs against conserved regions in the HIV-1 RNA genome (ter Brake, Mol. Ther., in press). Some of these shRNAs also target the lentiviral vector genome (Fig. 6a; LDR9, Pol29 and Nef19). We transfected 293T cells with the different shRNA-expression constructs and 24 hours later with the appropriate reporter constructs. Alternatively, we infected these cells after 24 hours with JS1-wtNef lentiviral vector. The 3 additional shRNAs demonstrated full inhibitory activity on the luciferase reporters (Fig. 6b; right 3 panels), but lacked any activity on the incoming RNA genome (Fig. 6c), with one notable exception: shNef19 is an effective inhibitor in both systems. The explanation for this exception comes from inspection of its target in the lentiviral vector genome (Fig. 6a), which is actually located in the 3'LTR region, and thus part of the GFP transcript. The observed drop in GFP-expressing cells is therefore caused by direct RNAi-inhibition of the reporter transcript, and not by targeting of the incoming RNA genome.

## Discussion

We have not observed RNAi-mediated targeting of the HIV-1 RNA genome of incoming particles using our lentiviral vector transduction system. The human T cell line that stably expresses shRNAs directed against the viral Nef gene shows effective inhibition of HIV-1 replication [24]. However, we could not demonstrate an effect on the level of transduction with lentiviral particles, pseudotyped either with VSV-G or wildtype HIV-1 envelope. Similar results were obtained in a cell line transiently transfected with an shNef-expressing plasmid prior to infection. The intracellular levels of shRNAs is much higher upon transfection than in stable cell lines (results not shown), but even this increased concentration did not seem to affect the transduction efficiency. In addition, we failed to obtain an inhibitory effect on the incoming RNA genome with other shRNAs that target different parts of the HIV-1 RNA genome or after transfection of a synthetic siRNA against Nef. All these results strongly indicate that the incoming HIV-1 RNA genome is not a target for RNAi.

The contradicting results that have been reported in literature may be due to differences in experimental conditions. It has been claimed that differences in target accessibility of different regions of the packaged RNA genome contribute to the variation in experimental results, but we detected a lack of inhibition with a range of targets, which are all highly accessible for RNAi-mediated inhibition in the context of reporter constructs. Furthermore, we demonstrated efficient targeting of the HIV-1 RNA genome in the producer cell, before it is encapsidated in the virion particle. It has been reported that the cellular environment can affect both the efficiency and the specificity of siRNAs and shRNAs [35]. The use of different cell types can influence the observed RNAi effect. Additionally, the use of different promoters in shRNA expression plasmids might also influence the potency of inhibition [36]. In addition, "nude siRNAs", not associated with RISC, may be able to enter the viral core when present at high concentrations. Subsequent binding to the viral RNA genome can induce antisense-mediated inhibition of reverse transcription, but not an RNAi effect.

An explanation for the absence of targeting of the incoming viral RNA genome is inaccessibility to the RNAi machinery. After fusion of viral particles with the target cell membrane, the virion core is released into the cytoplasm. This coneshaped core consists of the capsid (CA-p24) protein containing the RNA genome and viral enzymes. This core is dissolved only partially during the infection process. Furthermore, when the reverse transcription complex (RTC) is formed, the genomic RNA is still associated with multiple proteins (nucleocapsid [NC], reverse transcriptase [RT], matrix protein [MA] and integrase [IN]). The limited knowledge about the structure of intracellular retroviral complexes prohibits a detailed discussion, but there is supportive evidence that large molecules cannot enter the core particle in which reverse transcription occurs. For instance, it was shown that tRNA molecules can enter the core particle in virus-infected cells, but with an efficiency that is 4 to 5 orders of magnitude lower than the tRNA packaging efficiency in virion-assembling cells [37]. We made a similar observation with RNAi targeting the vector genome. During lentiviral vector production the RNA genome is an efficient target, resulting in reduced titers. In contrast, RNAi directed against the incoming genome could not reduce the transduction efficiency. Given the size of the RISC complex, it is likely that RISC cannot enter the viral particle, thereby explaining our results.

## Conclusion

Using lentiviral vector transduction as a model for HIV-1 infection, we have shown that the incoming HIV-1 genome cannot be targeted directly by RNAi. For effective gene therapy applications based on RNAi, it would be beneficial to target the incoming virus, thus blocking provirus establishment and in fact new infection of cells. To achieve this objective, one should target cellular receptors or co-factors that are involved in the initial phase of infection [15,38].

## Methods

### Plasmid construction

Lentiviral vector plasmids are derived from the construct pRRLcpptpgkgfppreSsin [39], which we renamed JS1. The plasmids JS1-Nef and JS1-R2 were obtained by digestion of the firefly luciferase expression vectors pGL3-Nef and pGL3-R2, containing an ~250-bp Nef fragment downstream of the luciferase gene [29], with XhoI and PstI and inserting this fragment into the corresponding sites of JS1. The other firefly reporter plasmids (pGL3-LDR9 and -Pol29 and -Nef19) were constructed by insertion of a 50–70 nucleotide HIV-1 sequence, with the 19-nucleotide target in the center, in the EcoRI and PstI sites of pGL3-Nef (ter Brake et al.; in press).

The pSUPER vector [8], which contains the H1 polymerase III promoter, was linearized with BglII and HindIII. Sense and antisense strand oligonucleotides, which encode the shRNA sequence against a conserved 19-nucleotide HIV-1 region (LDR9; AGATGGGTGCGAGAGCGTC [798], Pol29; CAGTGCAGGGGAAAGAATA [4811] and Nef19; GGGACTGGAAGGGCTAATT [9081] ter Brake et al.; in press) or the Nef [24] sequence, were annealed and ligated into pSUPER. The number between the brackets indicates the nucleotide position in prototype HIV-1 strain HXB2. The plasmid pRL-CMV (Promega) expresses Renilla luciferase under control of the CMV promoter.

### Cell culture

Human embryonic kidney (HEK) 293T adherent cells were grown at 37°C and 5% CO_2 _in DMEM (Gibco BRL) and SupT1 suspension cells were grown in RPMI 1640 (Gibco BRL), both supplemented with 10% Fetal Calf Serum (FCS), penicillin (100U/m) and streptomycin (100 μg/ml). The SupT1 cells stably expressing shNef were described previously [24].

### Lentiviral vector production

293T cells were grown to 50% confluence in 2 ml culture medium in 9.4 cm^2 ^wells. The medium was replaced with 2 ml medium without antibiotics. Subsequently, the lentiviral vector plasmid (2.2 μg) was co-transfected with packaging plasmids pMDLg/pREV (1.45 μg), RSV-REV (0.56 μg), and pVSV-G (0.78 μg) [40,41] or the pSV7D plasmid encoding HXB2 gp160 (0.78 μg). The pSV7D Envelope gp160 plasmid was a kind gift of Dr. J. Binley (Torrey Pines Institute for Molecular Sciences, La Jolla, CA, USA). Co-transfection in 3 ml was performed with 5 μl lipofectamine-2000 and 0.5 ml Optimem (Gibco BRL). The culture medium was refreshed after 16 hrs. Medium containing the lentiviral vector was harvested the next day and replaced with fresh medium. This procedure was repeated after 24 hrs. The supernatants were mixed, cellular debris was removed by low speed centrifugation and aliquots of 0.5 ml were stored at -80°C. For lentiviral vectors produced with HIV-1 envelope, the stocks were concentrated with an Amicon Ultra concentrator, MWCO 100,000 (Millipore Corporation, Bedford, MA, USA).

### Lentiviral vector transduction

Lentiviral vector stocks were titrated on 293T cells and SupT1 cells. SupT1 (1.0 × 10^5 ^cells in 0.5 ml medium) and 293T (1.0 × 10^5 ^cells in 0.5 ml medium) were subsequently transduced at various m.o.i. (from 0.01 to 1). Two days after transduction the cells were harvested, fixated in 4% paraformaldehyde and analysed by FACS for GFP expression (FACScan, BD Biosciences).

### Transfection experiments

293T cells (2 cm^2^; 1.0 × 10^5 ^cells) were seeded in 500 μl DMEM with 10% FCS without antibiotics. The next day, 1 μg pSUPER-shRNA plasmid, 125 nM siRNA or 1 μg control pBS (pBluescriptII (KS+); Stratagene) was transfected with 1 μl lipofectamine-2000 in a reaction volume of 100 μl according to the manufacturers instructions (Invitrogen). Sixteen hrs post-transfection the medium was replaced with 500 μl medium with antibiotics, and the cells were subsequently used for transduction or luciferase experiments.

For luciferase experiments, 293T cells (2 cm^2^; 60% confluent) were transfected with 200 ng pGL3-constructs and 1 ng pRL using lipofectamine-2000. SupT1 cells (shNef-expressing and control) were transfected with luciferase plasmids by electroporation. Briefly, 5 × 10^6 ^cells were washed in RPMI 1640 medium with 20% FCS and mixed with 5 μg pGL3-constructs and 150 ng pRL in 250 μl of RPMI 1640 medium with 20% FCS. Cells were electroporated in 0.4 cm cuvettes at 250 V and 975 μF and subsequently resuspended in RPMI 1640 medium with 10% FCS. The culture medium was refreshed after 16 h. After another 24 h, the cells were lysed in 150 ml of Passive Lysis Buffer (PLB) (Promega). Firefly and renilla luciferase activities in the lysate were measured with the Dual-luciferase Reporter Assay System (Promega).

## Competing interests

The author(s) declare that they have no competing interests.

## Authors' contributions

EMW participated in design of the study, carried out the transfection and transduction experiments and drafted the manuscript. OtB participated in conception and design of the study and carried out the lentiviral vector production experiments. BB participated in design and coordination of the study and helped to draft the manuscript.
